# Heterogeneous and Dynamic Prevalence of Asymptomatic Influenza Virus Infections

**DOI:** 10.3201/eid2405.160782

**Published:** 2018-05

**Authors:** Nancy H.L. Leung, Benjamin J. Cowling

**Affiliations:** The University of Hong Kong, Hong Kong, China

**Keywords:** epidemiology, influenza virus, viruses, asymptomatic infections, subclinical infections, influenza

**To the Editor:** We read with interest the article by Furuya-Kanamori et al. on the proportion of influenza virus infections that are asymptomatic or subclinical (*1*), and we are troubled by a series of fundamental flaws and errors. We were concerned that the authors presented pooled estimates of the asymptomatic fraction, given the massive heterogeneity in estimates (Ι^2^ values of 97%–98% in Technical Appendix Table 1). It is not considered good practice to present pooled estimates in instances of massive heterogeneity (*2*). We were very surprised that the authors included volunteer challenge studies because it is well known that the severity of these infections can be modulated by the route of administration and possibly the infectious dose. We also were surprised that human infections with avian influenza viruses were included because the epidemiology of these infections differs markedly from that of human influenza viruses. These studies were mistakenly labeled as studies of pandemic influenza in Technical Appendix Table 1. When reviewing serologic studies, the authors did not define a specific antibody titer threshold but relied on the choices made in individual studies; studies that inferred influenza virus infections based on low postepidemic hemagglutination-inhibition titers, such as 10 or 20, may lack specificity because some persons could have preexisting antibodies (*3*). Measurement error can also be a concern. The authors probably should have excluded such studies.

In another systematic review of the asymptomatic fraction of influenza virus infections (*4*), we found that study designs could explain a great deal of heterogeneity in the asymptomatic fraction in studies such as outbreak investigations that used molecular testing to confirm influenza virus infections rather than serologic studies that used antibody titer measurements to indicate infections. Asymptomatic fractions were higher in general, and much more heterogeneous, in studies that followed the latter approach.
